# Patterns of Dietary Supplement Use during the COVID-19 Pandemic in Poland: Focus on Vitamin D and Magnesium

**DOI:** 10.3390/nu16193225

**Published:** 2024-09-24

**Authors:** Patrycja Grosman-Dziewiszek, Izabela Jęśkowiak-Kossakowska, Adam Szeląg, Benita Wiatrak

**Affiliations:** Department of Pharmacology, Faculty of Medicine, Wroclaw Medical University, Mikulicza-Radeckiego 2, 50-345 Wroclaw, Poland; patrycja.grosman-dziewiszek@umw.edu.pl (P.G.-D.); adam.szelag@umw.edu.pl (A.S.); benita.wiatrak@umw.edu.pl (B.W.)

**Keywords:** vitamin D, dietary supplements, COVID-19, Poland, public health, immunity, magnesium

## Abstract

**Background:** The COVID-19 pandemic has brought significant attention to the role of dietary supplements, particularly Vitamin D, in enhancing immunity and possibly mitigating the severity of the disease. The pandemic has highlighted the importance of nutritional health in preventing severe outcomes from infections. **Objective:** This study aimed to assess consumption patterns of dietary supplements, with a focus on Vitamin D, among the Polish population during the COVID-19 pandemic and to identify the demographic factors influencing these patterns. **Methods:** An anonymous survey was conducted in March 2021 among 926 pharmacy patients in Poland. The study analyzed the use of dietary supplements such as vitamin D, magnesium, and others in relation to variables like age, gender, and education level. Statistical analyses were performed using the Pearson chi-square test. **Results:** The study revealed that 77.1% of the respondents reported using dietary supplements, with Vitamin D being the most frequently mentioned, used by 64.6% of participants. Magnesium was also widely used, with a higher overall prevalence of 67.3%, making it the most commonly consumed supplement. The use of supplements was significantly higher among women and individuals with higher education. Younger age groups, particularly those aged 18–30, were more likely to use supplements. **Conclusions:** The use of supplements was significantly higher among women, individuals with higher education, and those aged 18–30. However, the findings also indicate a growing awareness and increased use across the general population. This trend reflects increased public awareness of the potential health benefits of these supplements in boosting immunity. However, the study also highlights the need for public education on the risks of over-supplementation and the importance of appropriate dosages.

## 1. Introduction

Nearly five years after the first detection of the SARS-CoV-2 virus in Wuhan, China, in late December 2019, the COVID-19 pandemic remains a significant global health challenge [1]. Despite seasonal fluctuations in infection rates, the threat posed by the virus persists, and we still lack a complete understanding of why some individuals experience mild symptoms while others suffer severe complications or death. One of the major breakthroughs in combating COVID-19 has been the development of vaccines. However, vaccines do not provide complete protection against infection [1].

The World Health Organization (WHO) officially declared COVID-19 a pandemic on 11 March 2020 [2,3]. The disease affects nearly all organs of the human body, with its pathology involving mechanisms such as hypoxia, hemodynamic instability, vascular injury, and immune system overactivation, leading to excessive inflammation and cytokine storms, particularly in the respiratory system [4].

The severity of COVID-19 is influenced by various factors, including a person’s metabolic status, age, pre-existing medical conditions, nutritional status, immune function, and lifestyle. Notably, comorbidities such as type 2 diabetes, hypertension, and heart disease are significant risk factors for severe disease outcomes [5].

Traditional healthcare has been restricted due to the increasing number of new COVID-19 infections. Routine medical consultations and diagnostic services, including those for chronic diseases such as cardiovascular disease, diabetes, and cancer, have been severely disrupted [6]. As a result, telemedicine has rapidly developed to bridge the gap in healthcare accessibility. Telemedicine, including services such as teledermatology and telerehabilitation, has become essential to ensuring continuity of care for patients, allowing them to receive consultations and treatments remotely [6,7]. This change has also coincided with the trend of self-supplementation, where patients have tried to improve their health using commonly available vitamins and minerals [3], including vitamin D, magnesium, and other supplements, without direct medical supervision.

One factor that may exacerbate the risk of COVID-19 infection is compromised immunity. This study focuses on the impact of supplementation with vitamin D, vitamin C, magnesium, zinc, iron, omega-3 fatty acids, and fish oil on immunity and the progression of COVID-19. Among these, vitamin D has garnered considerable attention.

Vitamin D (1,25-dihydroxy vitamin D3) is a fat-soluble vitamin that is primarily synthesized in the skin upon exposure to sunlight, but it can also be obtained from foods such as oily fish, meat [8], milk, and fortified foods [9]. In some countries, e.g., Finland and the United Kingdom, food products such as liquid dairy products, breakfast cereals, and fat spreads are fortified with vitamin D to prevent deficiency [9,10]. Vitamin D deficiency is defined as a serum concentration of 25-hydroxyvitamin D (25(OH)D) between 25 and 50 nmol/L [10]. The studies have shown changes in vitamin D status across different life stages, with middle-aged individuals often experiencing significant shifts in serum 25-hydroxyvitamin D levels [10,11]. One study has reported the beneficial role of vitamin D supplementation in maintaining muscle strength, improving physical function, and decreasing the risk of falls among older people with low levels of serum vitamin D [12]. Vitamin D deficiency increases the risk of multiple sclerosis and epilepsy [13]. Moreover, it has been reported that low blood levels of vitamin D have been linked to an increased risk of colds, flu, chest infections, and acute respiratory infections [14]. An association between vitamin D deficiency and the risk of cancers, hypertension, cardiovascular events, musculoskeletal disorders, and migraine has also been demonstrated [13,15,16,17]. Also, neuropsychiatric disorders such as schizophrenia, dementia, multiple sclerosis, epilepsy, autistic spectrum disorder, or depression are linked to low levels of vitamin D [17,18,19,20,21]. During the COVID-19 pandemic and the restrictions, both negative and positive changes in diets were observed [18].

Magnesium, another critical nutrient, plays an essential role in the activation and metabolism of vitamin D. Magnesium is required for the conversion of vitamin D into its active form in the body, which is crucial for its effectiveness in supporting bone health and immune function. Magnesium deficiency can impair the efficacy of vitamin D, making it necessary to consider adequate magnesium intake alongside vitamin D supplementation. During the pandemic, many individuals turned to magnesium supplements, recognizing their importance in conjunction with vitamin D for overall health and disease prevention [22].

The COVID-19 pandemic and the associated restrictions led to both negative and positive changes in dietary habits. A critical question is whether deficiencies in vitamin D, vitamin C, magnesium, zinc, omega-3 fatty acids, and other nutrients could influence the risk of COVID-19 infection and the severity of the disease. Evidence suggests that vitamin D deficiency may indeed impact COVID-19 outcomes, making supplementation crucial, especially since many people have inadequate levels of this essential nutrient. Traditional sources of vitamin D, such as diet and sun exposure, are often insufficient to maintain optimal blood levels year-round [23]. The widespread availability and affordability of vitamin D and magnesium supplements, coupled with recommendations from healthcare providers, have led to increased use, particularly during periods of reduced sunlight in autumn and winter [24].

This study aims to determine which dietary supplements were most commonly used by patients during the COVID-19 pandemic, focusing on the usage patterns of vitamin D, magnesium, and other supplements. The analysis considers factors such as age, gender, and education to better understand who is most likely to use these supplements.

## 2. Materials and Methods

### 2.1. Study Design and Participants

An anonymous study in the form of an electronic and paper questionnaire was conducted in March 2021 among 926 pharmacy patients in Poland. Pharmacy patients in this study refer to individuals who visited pharmacies in Wrocław and other parts of Poland. The selection process was random, and the survey was distributed both in paper form and online across the country via Google^®^ Forms. Participation was voluntary and anonymous. The paper version of the survey was intended only for people who do not use electronic devices, and its use was very limited due to sanitary reasons during the COVID-19 epidemic.

The currently published research results are part of one joint project carried out simultaneously using the same survey. Some of the research results included in the survey regarding patients’ habits and the role of pharmacists and telemedicine during the COVID-19 pandemic have been developed and published. This paper presents an analysis of previously unreported and unpublished research results regarding vitamin D and dietary supplements in Poland during the COVID-19 pandemic. The study group in both studies included the same probands. The control group consisted of patients not using any supplements.

### 2.2. Ethical Approval

The study was performed following the ethical standards laid down in the 1964 Declaration of Helsinki and its later amendments. The study protocol was approved by the Institutional Review Board and Bioethics Committee of Wroclaw Medical University, Wroclaw, Poland (No: KB-253/2021), and the approval date is 18 March 2021.

### 2.3. Study Questionnaire

The questionnaire consisted of three sections. The first section of the survey explored demographic variables, including sex (male, female) and age (divided into groups 198–30, 31–40, 41–50, 51–60, 61–70, 71–80, and over 80 years of age) and education characteristics. The demographic variables included sex (male, female), age (divided into groups 19–30, 31–40, 41–50, 51–60, 61–70, 71–80, and over 80 years of age), and education (higher, secondary, primary, vocational, student). The content of the questionnaire also included medications taken for chronic disease, access to medical care, and preventive examinations performed during the COVID-19 pandemic.

### 2.4. Statistical Analysis

Differences between subgroups were calculated using the Pearson chi-square test using Statistica v13.0.

## 3. Results

### 3.1. Population Characteristics

We conducted a study among patients aged 18 and older. Data were collected from a total of 926 individuals. The study shows that 43% (*n* = 398) of respondents were aged 18–30, 20% (*n* = 185) were aged 31–40, 14.7% (*n* = 136) were aged 41–50, 10.5% (*n* = 97) were aged 51–60, 9.4% (*n* = 87) were aged 61–70, 1.6% (*n* = 15) were aged 71–80, and 0.9% (*n* = 8) were over 80 years old (Table 1). The elderly patients mostly did not use the internet and required a paper version of the survey, which posed a high risk during the COVID-19 pandemic. Of the respondents, 76.9% (*n* = 712) were women and 23.1% (*n* = 214) were men. Although more women participated in the study, the groups of women and men were analyzed separately in the statistical analysis. Regarding education level, the majority of participants had higher education (*n* = 552, 59.6%), 21.7% (*n* = 201) were students, 16.3% (*n* = 151) had secondary education, 0.6% (*n* = 6) had primary education, and 1.7% (*n* = 16) had vocational education.

### 3.2. Taking Dietary Supplements

**Figure 1:**
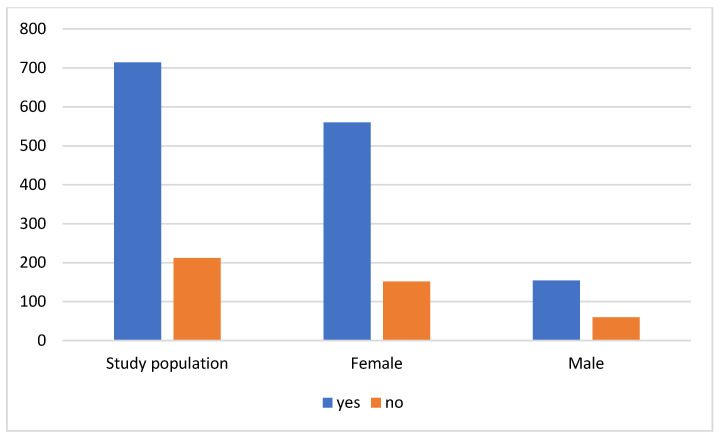
Taking dietary supplements.

Of the 926 individuals included in this study, 714 respondents (77.1%) take dietary supplements. Among them, 560 (78.4%) are women, and 154 (21.6%) are men (Figure 1). Statistical analysis revealed that a significantly greater number of respondents take dietary supplements than do not (*p* = 0.04), suggesting that taking dietary supplements is a common practice in the studied population. Moreover, a statistically significant number of people with higher education were found to take supplements more often than respondents with other education statuses (57.2%, *p* = 0.01).

The most commonly used supplement was vitamin D, which was taken by 598 people, representing 64.6% of the respondents. Among those surveyed, 328 individuals (35.4%) did not take this supplement. This result is statistically significant (*p* < 0.001), indicating a widespread use of vitamin D supplements in the studied population (Table 2).

Another frequently consumed supplement among the study participants was magnesium. It was used by 623 individuals, accounting for 67.9% of those surveyed (Table 2). This result was also statistically significant, suggesting that magnesium is a popular choice among supplements.

Other supplements were taken much less frequently. Vitamin C was used by 257 people, representing 27.8% of the respondents, while omega-3 was consumed by 158 individuals (17.1%). Multivitamin preparations were used by 132 people (14.3%), and zinc was used by 115 individuals (12.4%). Iron was taken by 10 people (1.1%), and vitamin B was taken by 28 (3.0%). The least popular supplements were iodine (2 people, 0.2%) and folic acid (10 people, 1.1%). Age-related analysis revealed that individuals aged 19–30 were significantly more likely to take vitamin B compared to older age groups (*p* < 0.05). Within the cohort of vitamin B users, 40.8% were aged 19–30.

**Figure 2:**
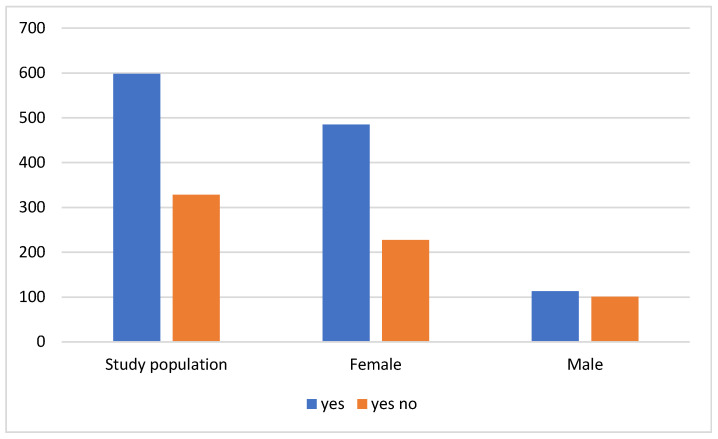
Taking vitamin D.

The statistical analysis of vitamin D supplementation revealed that 35.4% of the population did not use vitamin D supplements, while 64.6% did (*p* = 0.00004). Notably, women constituted the majority in both groups. Among men, 101 individuals (10.9% of the total) did not use vitamin D supplements, whereas 113 (12.2% of the total) did (Figure 2). This distribution was relatively balanced. In contrast, more than half of the surveyed women (485, or 52.4% of the total) reported using vitamin D supplements. The significant gender disparity in supplement use patterns highlights potential differences in health behaviors and/or medical advice received between men and women.

**Figure 3:**
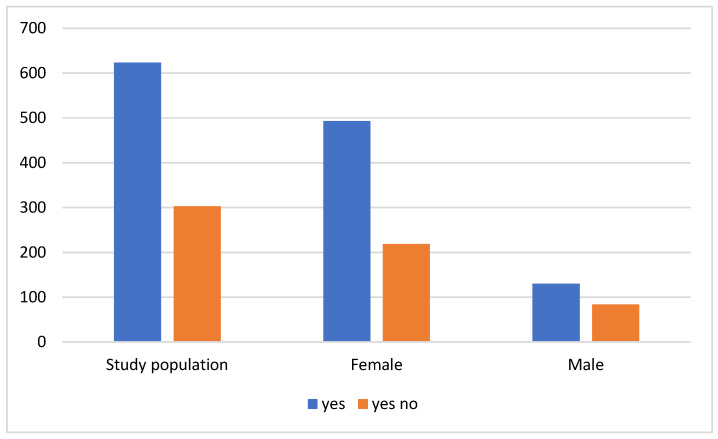
Taking magnesium.

Figure 3 illustrates the prevalence of magnesium supplementation within the studied population, highlighting its frequent use, particularly when compared to other supplements. Both men (60.7%) and women (69.0%) reported higher rates of magnesium supplementation than non-supplementation. Moreover, a statistically significant observation showed that older individuals take magnesium more often than younger individuals (*p* < 0.001).

## 4. Discussion

This study, conducted during the COVID-19 pandemic, surveyed 926 participants, the majority of whom were women (76.9%), with a significant number holding higher education degrees (59.6%). The age distribution showed that 43% of respondents were aged 18–30, with fewer participants in older age groups. This demographic skew toward younger and more educated individuals likely contributed to the high prevalence of dietary supplement use observed.

A notable 77.1% of the study population reported using dietary supplements, with vitamin D being the most common (64.6%). This widespread use reflects the public’s proactive approach to boosting immunity during the pandemic, likely driven by concerns that vitamin D might support their immune system rather than by an awareness of a deficiency [25]. The popularity of vitamin D aligns with existing literature emphasizing its critical role in immune function and its potential protective effects against respiratory infections, including COVID-19 [26]. Public health campaigns and media coverage likely further increased the awareness and usage of this supplement during the pandemic [2].

Magnesium was also a popular supplement, used by 67.28% of respondents. Its prevalence may be attributed to its benefits in stress reduction and muscle function, which were particularly relevant when mental and physical health challenges were posed by the pandemic [27].

Gender differences were also observed, with women being more likely than men to use dietary supplements (78.4% vs. 21.6%). This finding aligns with broader research indicating that women are generally more engaged in health-promoting behaviors [28], including supplement use. Additionally, a statistically significant correlation (*p* = 0.01) was found between higher education levels and supplement use, suggesting that education plays a role in the awareness and adoption of health-promoting behaviors, possibly due to better access to information about the benefits of dietary supplements. This observation is consistent with other studies showing that individuals with higher education levels are more likely to engage in preventive health measures [29], including supplement use. In a study conducted in Australian society, it was shown with statistical significance that young, well-educated women with a higher awareness of healthier lifestyles most often take probiotics [29].

Younger participants (aged 18–30) were more likely to use supplements compared to older age groups, potentially reflecting generational differences in health behaviors or greater exposure to health information through digital media. This trend highlights the need for targeted public health messaging to ensure that older populations, who are more vulnerable to COVID-19, receive adequate guidance on supplement use [30].

During the COVID-19 pandemic, there was an observed increase in vitamin D supplementation in several populations [23]. The high prevalence of vitamin D and magnesium use during the pandemic underscores a broader public health awareness of these nutrients’ potential benefits in supporting immune function, and this widespread use also raises concerns about the risks of over-supplementation, particularly with fat-soluble vitamins like vitamin D, where excessive intake can lead to toxicity. Complications related to vitamin D3 toxicity may manifest as neuropsychiatric symptoms, cardiovascular symptoms with heart block, or acute kidney damage [4,31,32]. Early symptoms of vitamin D toxicity involve gastrointestinal tract disorders such as constipation, diarrhea, nausea, and vomiting. However, symptoms that may appear within days or weeks of use include drowsiness, constant headaches, bone pain, muscle and joint pain, and irregular heartbeats. Vitamin D intake increases the level of 25[OH]D in plasma to concentrations exceeding the binding capacity of Vitamin D Binding Protein [33]. The most frequently reported causes of vitamin D poisoning in patients were manufacturing errors, patient overdose or prescribers, and combinations of these factors [34]. Therefore, although vitamin D can be beneficial, the risk of toxicity from excessive intake highlights the need for public education on appropriate dosages, particularly given the increasing reports of vitamin D toxicity during the pandemic, as noted in studies showing hypervitaminosis D among supplement users.

The right dosage of vitamin D, depending on one’s age, body weight, and health condition, is crucial for proper supplementation, and there is a need for better public education on appropriate dosages and the risks associated with high levels of supplementation. Pregnant women should receive detailed instructions on the dosage of vitamin D. It is also important to be aware of taking vitamin D in multi-ingredient preparations, such as dietary supplements for pregnant women and for seniors, as they often contain the appropriate daily dose of vitamin D and additional intake can cause an overdose. This highlights the importance of appropriate advice from a pharmacist preceded by a thorough pharmaceutical interview. This need is particularly evident based on the results of a retrospective analysis of cohort data from the Charleston Area Medical Center (CAMC), where serum 25(OH)D levels were analyzed in patients. In this study, we observed a significant increase in serum 25(OH)D levels during the COVID-19 pandemic compared to the pre-pandemic period. Simultaneously, in the entire population, there was an increase in the percentage of patients with hypervitaminosis D [31].

The popularity of vitamin D during the pandemic may be partially explained by its potential role in modulating immune responses. The vitamin D receptor (VDR), expressed on immune cells such as monocytes, dendritic cells, and macrophages, plays a crucial role in reducing the secretion of proinflammatory cytokines such as interleukin-6 (IL-6) and indirectly affecting C-reactive protein (CRP) levels [30,35]. Elevated levels of these inflammatory markers have been associated with worse outcomes in COVID-19 patients [36]. Observational studies have confirmed that serum vitamin D concentrations are inversely correlated with the incidence or severity of COVID-19, highlighting the importance of maintaining adequate vitamin D levels as a potential preventive measure [36].

Moreover, vitamin D deficiency has been linked with other risk factors for severe COVID-19, including type 2 diabetes mellitus, insulin resistance, and obesity [8,37]. Diabetic patients with COVID-19 are at higher risk of infection and mortality than non-diabetic individuals due to COVID-19-induced insulin resistance. This leads to chronic metabolic abnormalities that were not present before infection [38]. Dietary patterns are known to play a role in the risk of many diseases, and certain components of the diet are particularly important for chronic diseases, including insulinemia and inflammation [39].

In regions around Asia, studies have reported positive correlations between vitamin D levels and COVID-19 infection rates and mortalities, underscoring the need for monitoring and potentially supplementing vitamin D levels in at-risk populations [2,8].

Magnesium, involved in numerous metabolic and biochemical processes, also plays a role in the immune response. It is essential for vitamin D metabolism and activation, as enzymes necessary for this process are magnesium-dependent. Low magnesium levels have been associated with poor prognosis in older people with bacterial pneumonia, and altered magnesium levels are common in patients with hypertension, cardiovascular diseases, diabetes, and obesity—conditions that are risk factors for severe COVID-19. For the general population, magnesium supplementation may help prevent COVID-19 infection [40,41,42,43].

Additional supplements, such as vitamin C, have been explored for their potential beneficial effect on COVID-19 outcomes. However, clinical trials have not consistently demonstrated significant effects; meta-analyses have shown that vitamin C supplementation does not significantly decrease mortality or the length of hospitalization, though it may reduce in-hospital mortality in some cases [33,44].

Zinc deficiency is associated with various pathological conditions, including delayed wound healing and impaired immune function. Zinc plays a crucial role in cell proliferation, differentiation, apoptosis, and DNA, RNA, and protein synthesis [45]. Although zinc, in combination with standard antiviral therapy, showed a synergistic effect during COVID-19 infection by potentially inhibiting viral replication, a randomized clinical trial of high-dose zinc and ascorbic acid supplementation in 214 patients with confirmed COVID-19 infection found no significant difference in symptom duration [44,45,46].

Iron, vital for immune function, plays a role in immune cell activation [47,48]. However, unbound iron can generate reactive oxygen species (ROS), leading to cellular damage and inflammation. During a COVID-19 infection, the virus-induced hypoxia and inflammation can result in anemia and a reduced bioavailability of iron. Meta-analyses have shown that lower hemoglobin levels correlate with the severity of COVID-19 infection patient prognoses [49,50].

Iodine is essential for immune function, and despite global salt iodization efforts, iodine deficiency remains a concern. Animal studies have suggested that products with iodine complexes and ascorbic acid may have in vivo activity against COVID-19, with promising results showing early viral clearance [51,52,53,54]. However, a clinical trial examining the effect of iodine supplementation on COVID-19 outcomes has yet to be published [55].

Omega-3 fatty acids, known for their anti-inflammatory and immune-supporting effects, are under investigation for their potential benefits in COVID-19 treatment. Although clinical trials of high-dose, fish-oil lipid emulsions in hospitalized COVID-19 patients are ongoing, these supplements have not yet been registered for COVID-19 treatment [56,57,58,59].

Overall, a balanced diet is crucial for immune responses, and one’s nutritional status significantly influences the course of COVID-19. However, data on the nutritional support of COVID-19 patients remain limited. Insulin resistance, which has been identified as a significant factor in COVID-19 severity, is influenced by diet. Studies such as Govender et al. (2021) have demonstrated that dietary changes can help reduce insulin resistance, improving outcomes in diabetic and COVID-19 patients [37]. Dietary patterns have a major role in the prevention of chronic diseases, including those that exacerbate COVID-19 outcomes [38].

Our study focused solely on the examination of taking vitamins and mineral supplements in a general Polish population during the COVID-19 pandemic. However, it is worth remembering that, in Poland, vitamins and minerals are available as medicinal products and dietary supplements. In Poland, the Food and Nutrition Safety Act specifies that dietary supplements are one of the food groups, defined as products that contain concentrated amounts of vitamins or minerals or other substances that have a nutritional or other physiological effect.

Medicines are defined in the Pharmaceutical Law, which specifies that medicinal products have the ability to prevent or treat diseases. If a product meets the conditions for qualification as both a food and a medicinal product, only the provisions of the law relating to medicinal products apply [60,61]. In practice, it is often difficult for many people to distinguish whether a product is a medicine or a dietary supplement because vitamins and minerals are found in both. For this reason, in order to standardize and simplify terminology, the questionnaire employed in this study asked respondents about the increased intake of dietary supplements during the COVID-19 pandemic. Another limitation of our study concerned the low representation and response rate of the oldest age group. One potential explanation for this is the fact that this demographic group may have faced greater challenges in participating in the survey, particularly during the COVID-19 pandemic. The elderly population, being at a higher risk of severe outcomes from COVID-19, may have been more cautious about interactions, including participating in research studies. Additionally, older adults often have limited access to or familiarity with digital technologies, which could have restricted their ability to engage with the online survey.

Our findings suggest that while certain supplements like vitamin D may have supported immune function during the COVID-19 pandemic, the overall effectiveness of dietary supplements in preventing or treating COVID-19 remains uncertain. Further research is needed to establish clear guidelines for supplementation, particularly regarding dosages and the potential risks of over-supplementation. Additionally, public health strategies should focus on educating the public about the benefits and limitations of dietary supplements, ensuring that vulnerable populations, such as the elderly and those with pre-existing conditions, receive appropriate guidance.

## 5. Conclusions

This study sheds light on the prevalent use of dietary supplements, particularly vitamin D and magnesium, during the COVID-19 pandemic among the Polish population. The high rates of supplement use, especially among women and individuals with higher education, suggest women in Poland are inclined more toward self-care and preventive health measures during the health crisis. While the benefits of Vitamin D in enhancing immune responses and potentially mitigating the severity of COVID-19 have been widely recognized, the study also underscores the importance of balanced and informed supplementation to avoid the potential risks associated with an excessive intake. The findings call for targeted public health initiatives to ensure that supplement use is both effective and safe, particularly as the world continues to navigate the challenges posed by the COVID-19 pandemic.

## Figures and Tables

**Table 1:** The characteristics of the study group [3].

Characteristic	Number of Participants [*n*]	Percent [%]
Gender		
Female	712	76.9
Male	214	23.1
Education		
Higher education	552	59.6
Student	201	21.7
Secondary education	151	16.3
Primary education	6	0.6
Vocation	16	1.7
Age groups		
18–30	398	43.0
31–40	185	20.0
41–50	136	14.7
51–60	97	10.5
61–70	87	9.4
71–80	15	1.6
>80	8	0.9
	Gender [*n*]	
Age groups	Female	Male
18–30	314	84
31–40	145	40
41–50	105	31
51–60	73	24
61–70	61	26
71–80	7	8
>80	7	1

**Table 2:** Taking supplements.

Supplements	Takes (YES)	Takes (NO)	*p*
Vitamin D	598	328	<0.001
Vitamin C	257	669	0.55
Omega-3	158	768	0.35
Multivitamin preparations	132	795	0.54
Magnesium	623	303	0.02
Zinc	115	811	0.56
Iron	10	916	0.32
Vitamin B	28	898	0.26
Iodine	2	924	0.43
Folic acid	10	916	0.08

## Data Availability

The raw data supporting the conclusions of this article will be made available by the authors upon request.
